# Rejuvenation of neutrophils and their extracellular vesicles is associated with enhanced aged fracture healing

**DOI:** 10.1111/acel.13651

**Published:** 2022-06-03

**Authors:** Xin Zhang, Gurpreet Singh Baht, Rong Huang, Yu‐Hsiu Chen, Kristin Happ Molitoris, Sara E. Miller, Virginia Byers Kraus

**Affiliations:** ^1^ Duke Molecular Physiology Institute, Duke University School of Medicine Duke University Durham North Carolina USA; ^2^ Department of Orthopaedic Surgery, Duke University School of Medicine Duke University Durham North Carolina USA; ^3^ Department of Pathology Duke University Medical Center Durham North Carolina USA; ^4^ Center for Electron Microscopy and Nanoscale Technology, Duke University School of Medicine Duke University Durham North Carolina USA; ^5^ Department of Medicine, Duke University School of Medicine Duke University Durham North Carolina USA

**Keywords:** age, extracellular vesicles, fracture healing, immune cells, immunosenescence, inflammaging, parabiosis, resilience

## Abstract

Tissue repair is negatively affected by advanced age. Recent evidence indicates that hematopoietic cell‐derived extracellular vesicles (EVs) are modulators of regenerative capacity. Here, we report that plasma EVs carrying specific surface markers indicate the degree of age‐associated immunosenescence; moreover, this immunosenescence phenotype was accentuated by fracture injury. The number of CD11b^+^Ly6C^intermediate^Ly6G^high^ neutrophils significantly decreased with age in association with defective tissue regeneration. In response to fracture injury, the frequencies of neutrophils and associated plasma EVs were significantly higher in fracture calluses than in peripheral blood. Exposure of aged mice to youthful circulation through heterochronic parabiosis increased the number of neutrophils and their correlated Ly6G^+^ plasma EVs, which were associated with improved fracture healing in aged mice of heterochronic parabiosis pairs. Our findings create a foundation for utilizing specific immune cells and EV subsets as potential biomarkers and therapeutic strategies to promote resilience to stressors during aging.

## Graphical abstract

Aged mice exhibited an immunosenescence phenotype that was accentuated by the physical stressor of fracture injury and was associated with defective tissue regeneration. Compared to solitary aged mice, the provision of youthful blood components by heterochronic parabiosis was associated with increased number of neutrophils in both peripheral blood and fracture calluses, and their associated plasma EVs, which may contribute to improved rejuvenation and bone fracture healing.

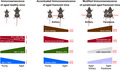

## 1 INTRODUCTION

Advanced age is associated with diminished tissue regeneration and leads to increased healthcare costs. Between 2000 and 2025, the population aged 50 years or older is predicted to increase by 60% in the United States, leading to an increasingly frail population that is at a greater risk of falls and fracture (Burge et al., 2007). A key factor to *successful aging—*defined by MacAuthur et al as freedom from disease and disability, high cognitive and physical functioning, and active engagement with life (Martin et al., 2015)—is the capacity for physical resilience. Physical resilience is the ability to resist or recover from functional decline following physical or health stressors (Whitson et al., 2016).

Although resilience at the tissue level is largely attributed to tissue‐resident stem cells involved in tissue maintenance and repair, recent evidence identifies extracellular vesicles (EVs) produced by stem cells as mediators of regenerative capacity (Wiklander et al., 2019; Zhai et al., 2020). EVs are microscopic particles, produced by all cells in the body, which circulate in the trillions in every milliliter of blood (Johnsen et al., 2019). Generally, exosomes (small‐sized EVs, SEVs) and microvesicles (medium‐sized EVs, MEVs) are generated from healthy cells and mediate intercellular communication and immune regulation, while apoptotic bodies (large‐sized EVs, LEVs) are released as a product of apoptotic cell disassembly and cell death and are becoming recognized as key players in the process of apoptotic cell clearance (Atkin‐Smith et al., 2015; Caruso & Poon, 2018). EVs carry surface markers derived from their parent cells, which can serve as a “fingerprint” to indicate their cellular origin, and carry internal cargo (proteins, lipids, RNA, mitochondria, and cytokines) capable of cell‐to‐cell communication and paracrine effector functions. The number, surface marker composition, and cargo content of EVs can reflect the physiological and pathophysiological condition of the body, including immune function changes during aging (Zhang et al., 2020; Zhang, Hsueh, et al., 2021; Zhang, Huebner, et al., 2021). EVs therefore have the potential to be “direct” biomarkers in the causal pathway of aging and resilience; that is, they can be both indicators and effectors of health outcomes (Kraus, 2018).

Bone fracture healing progresses through four overlapping stages involving inflammation, development of a cartilaginous callus, development of a boney callus, and remodeling of the healing tissue (Marsell & Einhorn, 2011). Given that hematopoietic cells, especially immune cells, are known to play an important role in fracture healing (Baht et al., 2018; Huang et al., 2020; Kovtun et al., 2016), we evaluated the responses of these cells and their associated EVs to the stress of tibial fracture injury. We previously reported that exposure to youthful circulation by heterochronic parabiosis improved fracture healing of aged mice. Using bone marrow transplantation and tissue culture models, we established that this rejuvenation is rooted in the secretome produced by young hematopoietic cells engrafted in the aged animal (Baht et al., 2015). For this reason, we evaluated cell and EV phenotypes, with and without heterochronic parabiosis, to identify potential promoters of resilient responses to stress. We utilized our murine tibial fracture model to identify specific immune cell subsets and their associated EVs that indicate and/or result in the observed differential capacity for fracture healing in young and aged mice. We hypothesized that specific immune cell and EV subpopulations change in an age‐dependent manner in response to fracture injury. To evaluate the profile of EV subsets in mice, we applied our recently developed high‐resolution multicolor flow cytometry methodology (Zhang et al., 2020; Zhang, Huebner, et al., 2021) to investigate the parent cells and companion EVs of all sizes using 12 surface markers representing the major murine hematopoietic cell types in peripheral blood.

## 2 RESULTS

### 2.1 Age‐associated decline in T cells and NK cells

**FIGURE 1:**
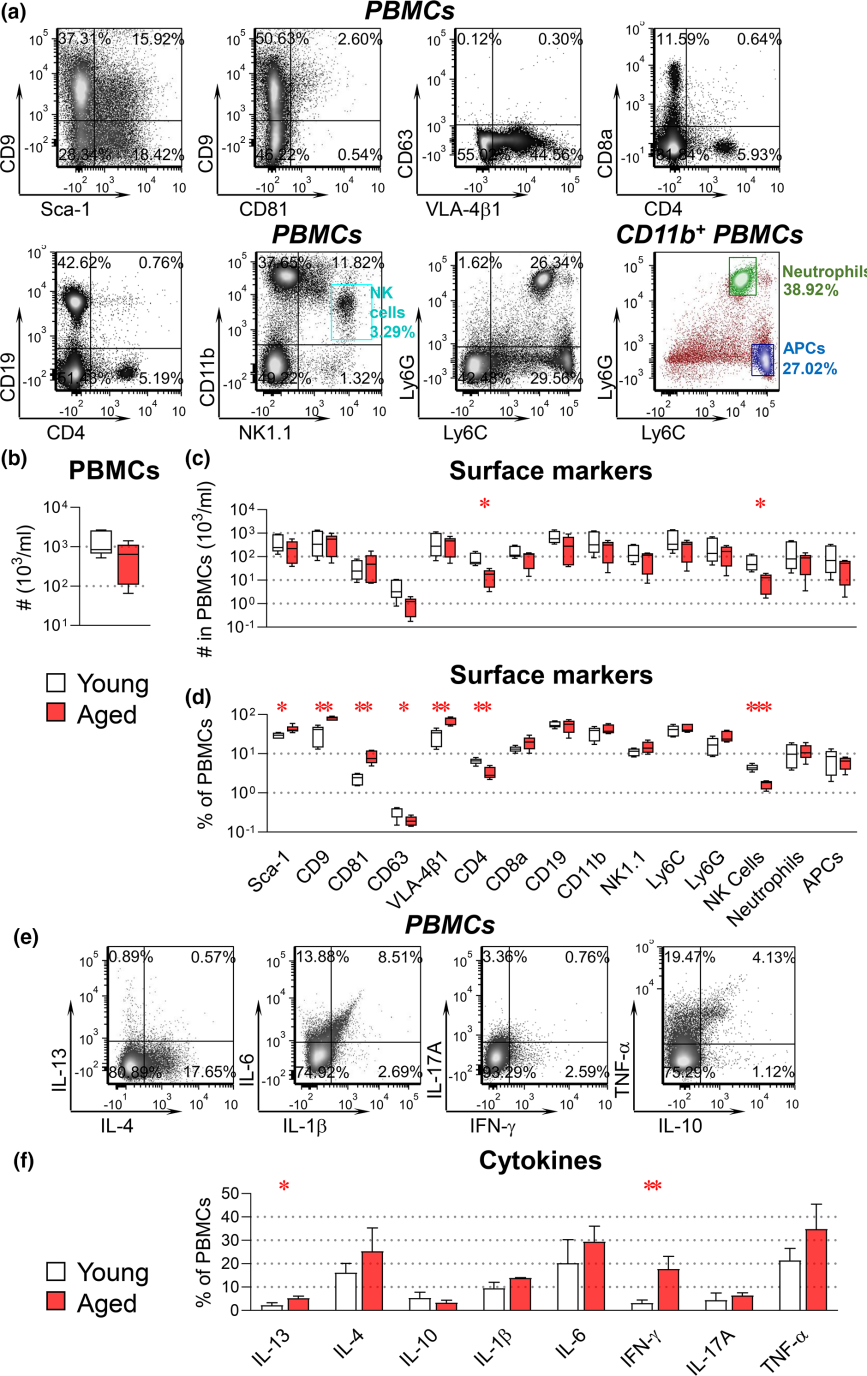
Aged mice exhibit an immunosenescence phenotype. PBMCs were derived from intact young (4 months old) and aged (24 months old) mice for profiling surface markers and intracellular cytokines. PBMCs were stained with fluorescence‐conjugated antibodies against the indicated surface markers (a–d). For intracellular cytokine staining (e–f), PBMCs were fixed, permeabilized, and stained with fluorescence‐conjugated antibodies against the indicated cytokines. The percentages and number of PBMCs carrying each tested molecule were determined by high‐resolution multicolor flow cytometry. Combination markers were used to define the following cells: CD11b^+^NK1.1^+^ for NK cells, CD11b^+^Ly6C^high^Ly6G^−^ for APCs, and CD11b^+^Ly6C^intermediate^Ly6G^high^ for neutrophils. (a) Representative dot plots present results of all tested surface markers in PBMCs or gated CD11b^+^ PBMCs. The graphs present a summary of the number of total PBMCs (b), as well as the number (c), and percentages (d), of PBMC subsets expressing each surface marker. (e) Representative dot plots present results of all tested cytokines in PBMCs. (f) Summary of the percentage of PBMCs carrying each cytokine. Comparisons between young and aged mice by an unpaired *t* test for each marker were performed with results indicated as *p < 0.05, **p < 0.01, and ***p < 0.001. (*n* = 5 per group)

Given the critical role of hematopoietic and particularly immune cells in age‐dependent shortcomings in fracture repair, we first investigated the blood of aged (24 months old) and young (4 months old) uninjured (intact) mice using a panel of 12 surface markers representing all major murine hematopoietic cell types (Table 1; Goh & Huntington, 2017; Lee et al., 2013; Mayle et al., 2013; Zhang et al., 2019; Zhang, Huebner, et al., 2021). Combination markers were used to define the following cell subsets: CD11b^+^NK1.1^+^ for NK cells, CD11b^+^Ly6C^high^Ly6G^−^ for antigen‐presenting cells (APCs) including monocytes, macrophages, and dendritic cells; and CD11b^+^Ly6C^intermediate^Ly6G^high^ for neutrophils (Table 1). Using high‐resolution multicolor flow cytometry, all tested markers were detected on the surface of murine peripheral blood mononuclear cells (PBMCs; Figure 1a). Lower total numbers of PBMCs were identified in aged than in young mice but did not reach statistical significance (Figure 1b); however, the number of CD4^+^ T cells and CD11b^+^NK1.1^+^ NK cells was significantly lower in aged mice (Figure 1c). Aged mice also had significantly lower percentages of CD4^+^ T cells, CD11b^+^NK1.1^+^ NK cells, and CD63^+^ cells, but significantly higher percentages of Sca‐1^+^, CD9^+^, CD81^+^, and VLA‐4β1^+^ cells (corresponding to hematopoietic stem cells [HSCs] and some immune cells; Figure 1d). Interestingly, aged mice also had higher percentages of IFN‐γ^+^ and IL‐13^+^ cells than young mice (Figure 1e,f). Taken together, these results suggest an immunosenescence phenotype in aged mice that may result from a deficiency in hematopoietic stem cell differentiation into terminal lymphoid cells (Rossi et al., 2005).

**TABLE 1:** Tested surface markers and their major expressing cells in blood circulation

Surface markers	Major cell origin
Sca‐1 (also known as Ly6A/E)	HSCs, B cells, T cells, dendritic cells
CD81	HSCs, B cells, T cells, NK cells, APCs
CD9	HSCs, B cells, T cells, NK cells, APCs
CD63	T cells, NK cells, platelets, basophils
VLA‐4β1 (also known as CD29)	T cells, B cells, NK cells, APCs, neutrophils
CD8a	Cytotoxic T cells
CD4	Helper T cells
CD19	B cells
CD11b	Neutrophils, monocytes, matured NK cells
NK1.1	NK cells (CD11b^+^NK1.1^+^)
Ly6C	APCs (CD11b^+^Ly6C^high^Ly6G^−^)
Ly6G	Neutrophils (CD11b^+^Ly6C^intermediate^Ly6G^high^)

Abbreviations: APCs, antigen‐presenting cells (including monocytes, macrophages, and dendritic cells); HSCs, hematopoietic stem cells; NK cells, natural killer cells; Sca‐1, stem cell antigen‐1; VLA‐4, very late antigen‐4.

### 2.2 Age‐dependent alterations of hematopoietic cell‐derived EV profiles

Using healthy human samples, we recently reported age‐associated declines in several subtypes of immune cell‐related plasma EVs (especially MEVs and SEVs). Furthermore, we identified a decrease in active (respiring) mitochondrial cargo within these populations of EVs, suggesting mitochondrial dysfunction‐induced immunosenescence of parent cells (Zhang et al., 2020). Therefore, a major goal of our current study was to address the relationship between parent cells and companion EVs using 12 surface markers representing the major murine hematopoietic cell types in peripheral blood.

**FIGURE 2:**
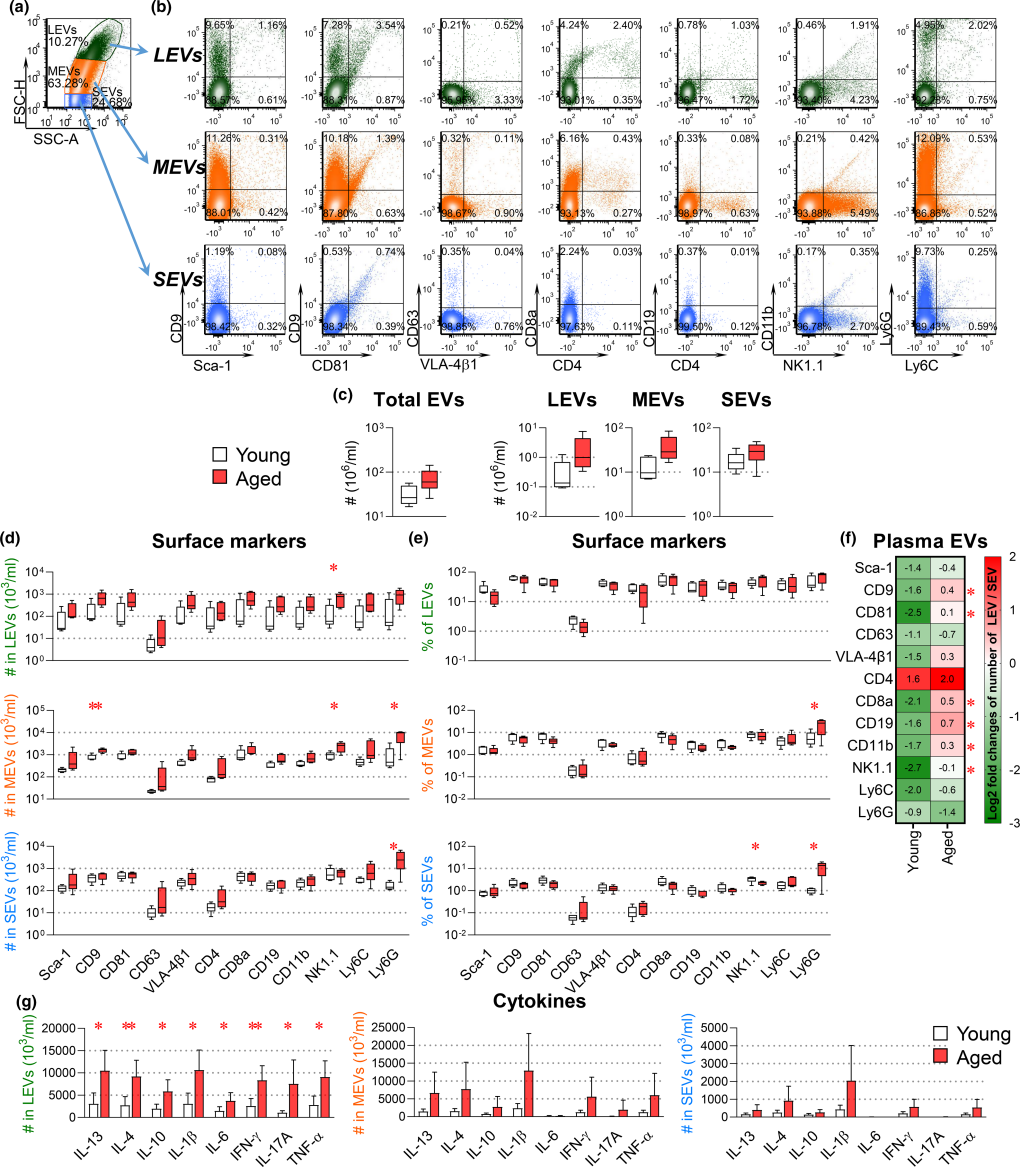
Age‐dependent changes in plasma EV surface markers of hematopoietic cells. Plasma EVs from young and aged mice were profiled for surface markers and intravesicle cytokines. EVs were stained with fluorescence‐conjugated antibodies against the indicated surface markers (a–f). For intravesicle cytokine staining (g), EVs were fixed, permeabilized, and stained with fluorescence‐conjugated antibodies against the indicated cytokines. The percentages and number of EVs carrying each tested molecule were determined by high‐resolution multicolor flow cytometry. (a) Representative color dot plots present size and granularity of LEVs, MEVs, and SEVs. (FSC‐H, forward‐scatter height; SSC, side‐scatter area.) (b) Representative color dot plots present results of all tested surface markers in gated individual plasma EV subsets. The graphs present a summary of the number of total EVs, LEVs, MEVs, and SEVs (c), as well as the number (d) and percentages (e) of EV subpopulations carrying each surface marker. (f) Heat maps presents the relative proportions of numbers of LEV to SEV (LEV/SEV) carrying the same surface markers within young and within aged mice. Positive (+) log2 fold changes of LEV/SEV represent more LEV than SEV, while negative (−) log2 fold changes represent fewer LEV than SEV. The SEV/LEV ratios differ between young and aged mice for seven EV subsets (indicated by *). (g) The graphs present a summary of the number of LEVs, MEVs, and SEVs carrying each cytokine. Comparisons between young and aged mice by an unpaired t test for each marker were performed with results indicated as *p < 0.05 and **p < 0.01. (*n* = 5 per group)

To profile signatures of murine hematopoietic EVs in mice, we applied our previously established method using high‐resolution multicolor LSRFortessa X‐20 flow cytometry and fluorescently labeled reference beads of various sizes (mean diameter range of 100–6000 nm; Zhang et al., 2020; Zhang, Huebner, et al., 2021). Consistent with observations in human plasma that were validated by dynamic light scattering (Zhang et al., 2020; Zhang, Huebner, et al., 2021), we identified three major subsets of EVs based on size (LEVs 1000–6000 nm, MEVs 100–1000 nm, and SEVs <100 nm) in murine plasma (Figure 2a). Notably, as discussed in our previous study (Zhang et al., 2020), the actual size of biological vesicles and calibration beads can differ as a result of multiple factors including but not limited to the differential composition of the EVs and beads, their different refractive indices, and the forward scatter collection angles of the flow cytometer. Therefore, our reported EV sizes represent approximations, not actual sizes. Nevertheless, the relative sizes of EV subsets (small to large) are valid, and calibration beads are useful tools for estimating the forward scatter‐based size of nano‐sized vesicles by flow cytometry. Transmission electron microscopy (TEM) imaging confirmed the size diversity of the separated EVs (Figure S1a). Consistent with the known presence of lipoproteins in EV preparations obtained by polymer‐based precipitation and ultrafiltration (Carnino et al., 2019), we detected small amounts (<0.5%) of ApoA1^+^ vesicles (Figure S1b). To limit the impact of contaminants on our observations, we only focused on EVs with the tested surface markers indicating their cell origins.

All tested hematopoietic markers were detected on the surface of murine plasma EVs by high‐resolution multicolor flow cytometry (representative staining Figure 2b). For each EV marker, we determined the percentage and number across the gated LEV, MEV, and SEV subtypes. In striking contrast to the data at the cellular level, we observed an age‐associated increase in the number of total EVs and EV subsets of all sizes (Figure 2c). Moreover, the number of NK1.1^+^ LEVs and MEVs, Ly6G^+^ MEVs and SEVs, and CD9^+^ MEVs (Figure 2d), as well as the percentages of Ly6G^+^ MEVs and SEVs (Figure 2e), was significantly higher, while the percentage of NK1.1^+^ SEVs (Figure 2e) was significantly lower in aged mice. The relative proportions of LEV to SEV (LEV/SEV) with the same surface markers were generally higher in aged than in young mice, especially those associated with immune cells (Figure 2f). Taken together, these data suggest that immunosenescence is manifested by age‐related changes in hematopoietic‐derived EV profiles. Furthermore, it appears that the nature of these changes in EV profile differs by the size of EV.

To better understand age‐related functional differences of EV subtypes, we quantified their expression of intravesicular cytokines, including IL‐13, IL‐4, IL‐10, IL‐1β, IL‐6, IFN‐γ, IL‐17A, and TNF‐α. Notably, the number of plasma EVs of all sizes carrying the tested pro‐inflammatory cytokines was generally higher in aged mice than in young mice, with statistical significance in LEVs (Figure 2f). These results are consistent with previous reports of associations between immunosenescence, pro‐inflammatory factors, and inflammaging (Fulop et al., 2017).

### 2.3 Age‐associated deficits in fracture healing are accompanied by an accentuated immunosenescence phenotype

**FIGURE 3:**
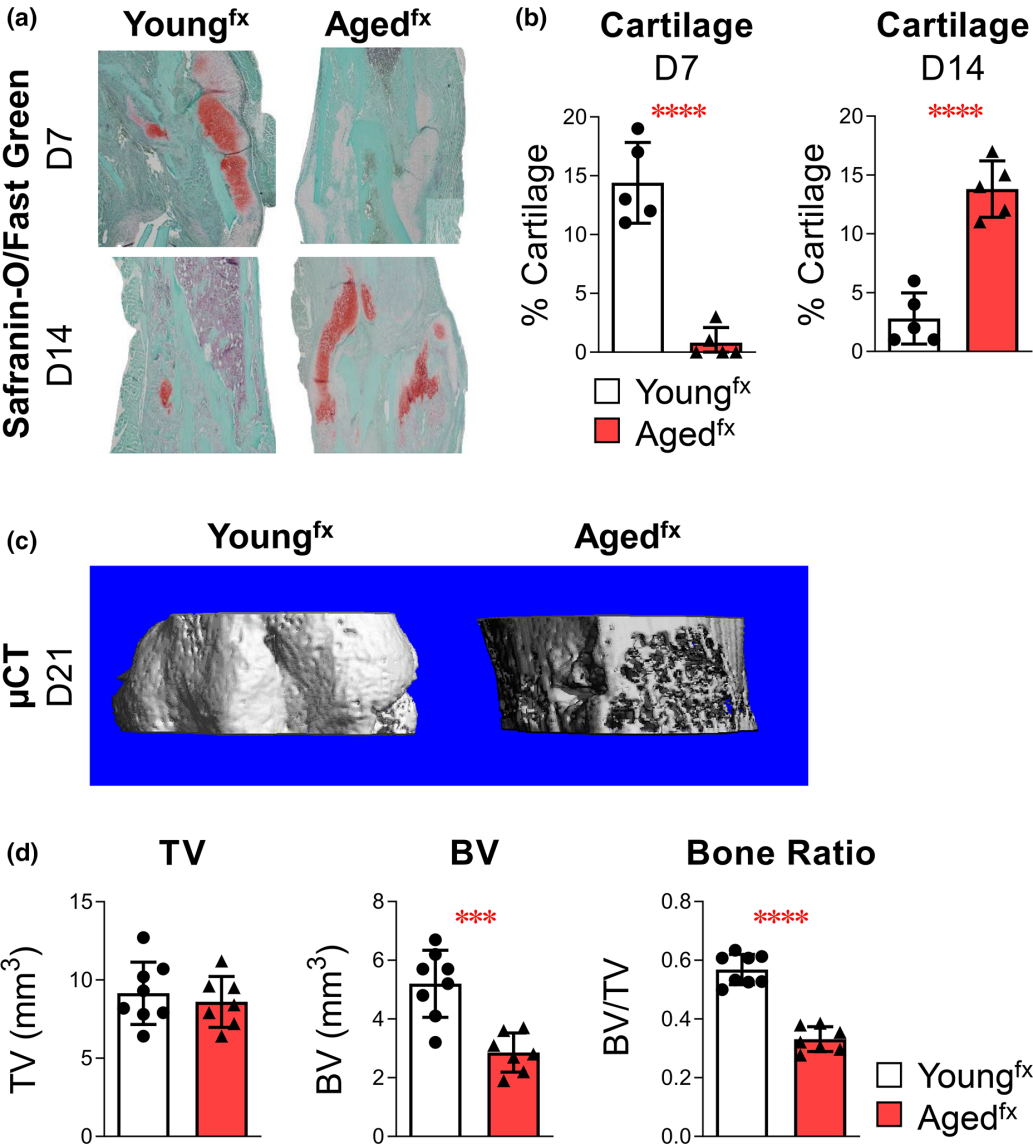
Bone fracture healing/tissue regeneration is delayed with advanced age. Young (4 months old) and aged (24 months old) mice underwent tibial fracture surgery. (a) Cartilage deposition was assessed by sectioning and staining 7‐ and 14‐day paraffin‐embedded fracture calluses with Safranin‐O/Fast Green. (b) The amount of cartilaginous tissue was determined using computer‐assisted histomorphometry. (c, d) Bone healing was assessed 21 days after injury using μCT analysis to measure TV, BV, and relative bone ratio (BV/TV). Cartilage analysis: young^fx^, *n* = 5 and aged^fx^, *n* = 5 mice. Bone analysis: young^fx^, *n* = 8 and aged^fx^, *n* = 7 mice. Data are expressed as mean ± standard deviation. Comparisons between young^fx^ and aged^fx^ mice by an unpaired t test for each marker were performed with results indicated as ***p < 0.001 and ****p < 0.0001

To understand the impact of age‐related immunosenescence on physical resilience, we used our well‐established murine tibial fracture model as a stressor (Baht et al., 2015; Vi et al., 2018; Xiong et al., 2018), and compared cellular and EV responses in young and aged mice. During the early stages of fracture healing, cartilage deposition was delayed in aged mice (Figure 3a,b). We confirmed delayed bone deposition 21 days post‐injury in the tibial fracture sites of aged fractured (^fx^) mice (24 months old) compared with young^fx^ mice (4 months old). Although the total volume (TV) of fracture calluses from young^fx^ and aged^fx^ mice was similar (Figure 3c,d), the bone volume (BV, Figure 3d) and the BV relative to TV (BV/TV, Figure 3d) within the fracture calluses of aged^fx^ animals were significantly lower.

To understand the involvement of immune cells in fracture healing, we compared the frequencies of all major immune cells in peripheral blood to the frequencies found within the fracture calluses 7 days after fracture. Relative to peripheral blood, fracture calluses contained significantly higher frequencies of CD11b^+^Ly6C^intermediate^Ly6G^high^ neutrophils, cells carrying individual neutrophil markers (CD11b, Ly6C, and Ly6G), and NK1.1^+^ cells; in contrast, compared with peripheral blood, fracture calluses contained significantly lower frequencies of CD4^+^ T cells and CD19^+^ B cells in both young^fx^ and aged^fx^ mice (Figure S2a,b). These findings agree with published data indicating a critical role for immune cells, especially neutrophils, in fracture healing (Bastian et al., 2016; Kovtun et al., 2016). Consistent with the findings of parent cell frequencies, the frequencies of multiple neutrophil markers were higher in fracture calluses than in plasma including IL‐11b^+^ MEVs and SEVs and Ly6C^+^ EVs of all sizes in young^fx^, and CD11b^+^ EVs of all sizes in aged^fx^ mice (Figure S3a,b).

**FIGURE 4:**
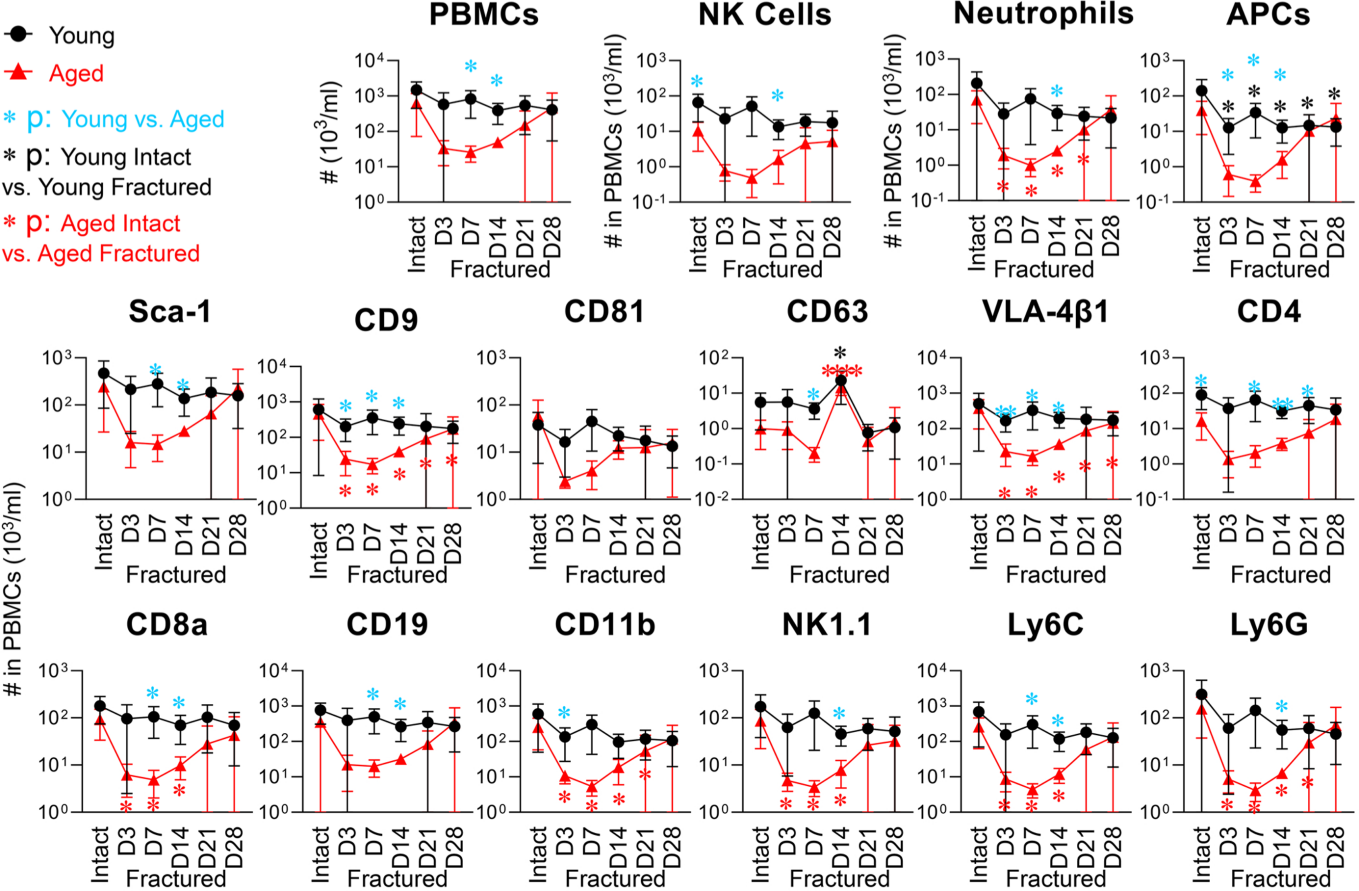
Immunosenescence phenotype in aged mice was accentuated by the physical stressor of fracture. PBMCs were derived from intact young (4 months old) and aged (24 months old) mice (*n* = 5 per group) and fractured mice (on Day [D]3, D7, D14, D21, and D28 post‐fracture, *n* = 4 in each group at each time point, except *n* = 6 in aged mouse group on D28). Following staining with fluorescence‐conjugated antibodies against the indicated surface markers, the number of PBMCs expressing each tested marker was determined by high‐resolution multicolor flow cytometry. The graphs present a summary of the number of total PBMCs, CD11b^+^NK1.1^+^ NK cells, CD11b^+^Ly6C^intermediate^Ly6G^high^ neutrophils, CD11b^+^Ly6C^high^Ly6G^−^ APCs, and PBMCs expressing each surface marker in young and aged intact and fractured mice. Comparisons between young and aged mice at each condition were performed using an unpaired t test. Comparisons between intact mice and fractured mice at each time point were performed using one‐way ANOVA with Holm–Sidak's multiple comparisons test (light blue asterisk: young vs. aged; black: young intact vs. young fractured; and red: aged intact vs. aged fractured): *p < 0.05, **p < 0.01, and ****p < 0.0001

**FIGURE 5:**
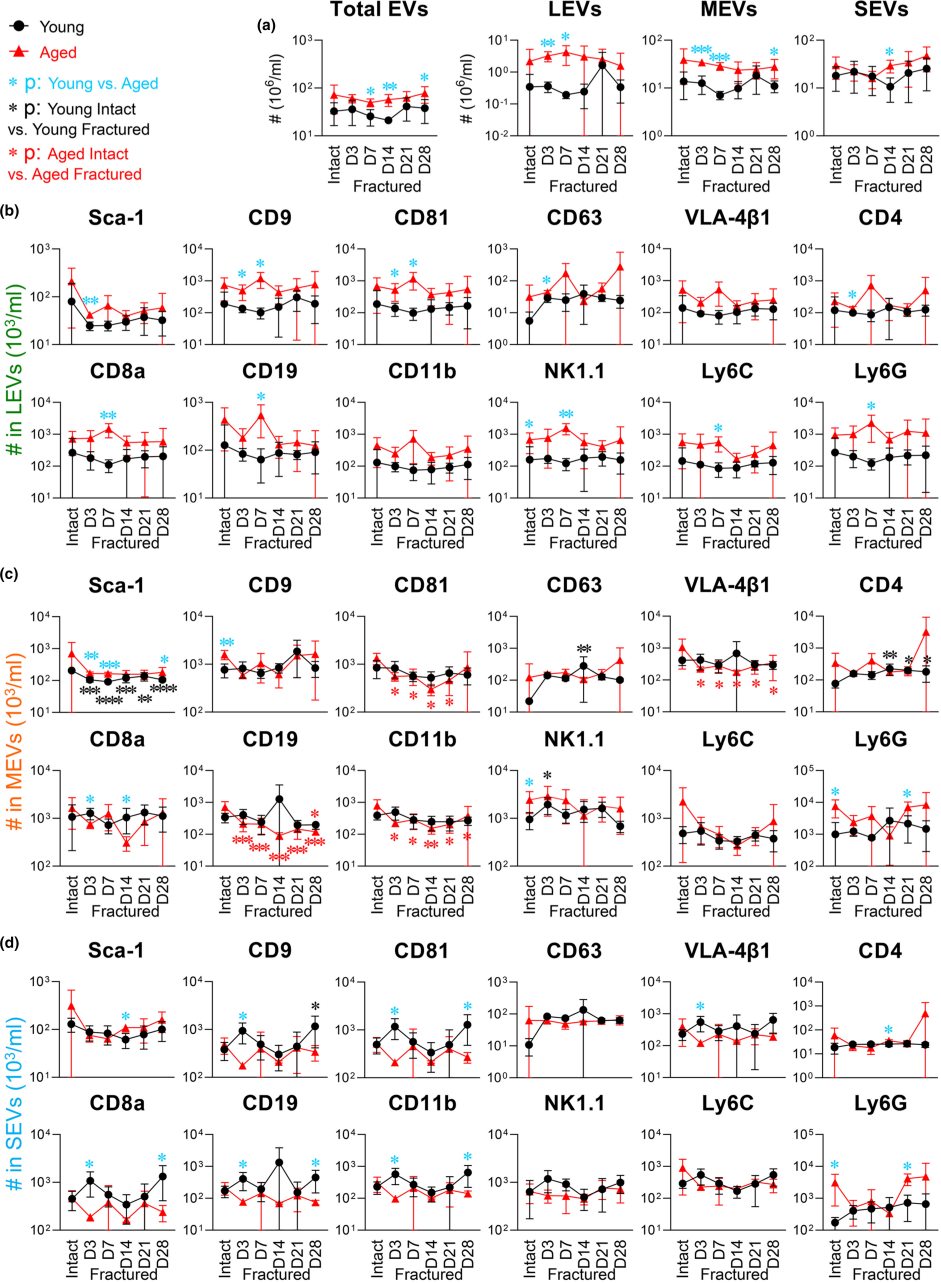
Post‐fracture variation of hematopoietic cell‐associated plasma EVs in young and aged mice. Separated plasma EVs from the same cohort of young and aged intact mice and fractured mice profiled for PBMCs in Figure 4 were stained with fluorescence‐conjugated antibodies against the indicated surface markers. The number of EVs expressing each tested marker in the gated LEVs, MEVs, and SEVs was determined by high‐resolution multicolor flow cytometry. The graphs present a summary of the total number of EVs, LEVs, MEVs, and SEVs (a), as well as individual subtypes of LEVs (b), MEVs (c), or SEVs (d), carrying each surface marker in young and aged intact and fractured mice. Young and aged mice were compared at each time point by an unpaired t test. Within young or aged (intact and fractured mice at each time point), comparisons were performed by one‐way ANOVA with Holm–Sidak's multiple comparisons test (light blue asterisk: young vs. aged; black: young intact vs. young fractured; and red: aged intact vs. aged fractured): *p < 0.05, **p < 0.01, ***p < 0.001, and ****p < 0.0001

To evaluate hematopoietic cells and their associated EVs in response to fracture, we profiled PBMCs and plasma EVs from young^fx^ and aged^fx^ mice on Day (D)3, D7, D14, D21, and D28 post‐fracture with the aforementioned 12 surface markers of the major murine hematopoietic cells (Table 1). In response to fracture within young^fx^ mice, there were several notable findings: The number of total PBMCs and most subsets of PBMCs did not significantly change; the number of APCs significantly decreased and remained low; and CD63^+^ cells significantly increased on D14 post‐fracture (Figure 4). In addition, Sca‐1^+^ EVs declined post‐fracture in young^fx^ mice (significant for young MEVs at all times; Figure 5b–d). Compared with young uninjured (intact) mice, young^fx^ mice had significantly higher numbers of NK1.1^+^ MEVs on D3, CD63^+^ MEVs on D14, CD4^+^ MEVs on D14‐28, and CD9^+^ SEVs on D28 (Figure 5c,d). Taken together, these results suggest that fracture injury in young mice triggers secretion of MEVs and SEVs by immune cells.

In response to fracture injury, the number of circulating immune cells of all types was reduced post‐fracture in aged^fx^ mice relative to young^fx^ mice; this change was significant for neutrophils, CD9^+^, VLA‐4β1^+^, CD8a^+^, CD11b^+^, NK1.1^+^, Ly6C^+^, and Ly6G^+^ cells for one or more time points, especially on D3‐14 post‐fracture, but not significant for CD81^+^ cells (Figure 4). Most cell counts in aged^fx^ mice recovered by D28 to the levels of aged uninjured mice, with the exception of CD9^+^ and VLA‐4β1^+^ cells, which remained low. Compared with young^fx^ mice, the numbers of all cell types were significantly lower in aged^fx^ mice post‐fracture at one or more time points (with the exception of CD81) consistent with an accentuated immunosenescence phenotype (Figure 4). In contrast to the parent cells, aged^fx^ mice had significantly more circulating total EVs, LEVs, and MEVs than young^fx^ mice at multiple time points post‐fracture (Figure 5a). Interestingly, whereas young^fx^ mice had a spike in several SEV subtypes (CD9^+^, CD81^+^, VLA‐4β1^+^, CD8a^+^, CD19^+^, and CD11b^+^) acutely on D3 post‐fracture, there was instead a decline of these SEV subtypes on D3 post‐fracture in aged^fx^ mice, demonstrating a significant age‐related difference in the acute response to fracture for these SEV subtypes (Figure 5d).

### 2.4 Youthful blood supply increases the number of neutrophils and their associated MEVs and SEVs in aged fractured mice

**FIGURE 6:**
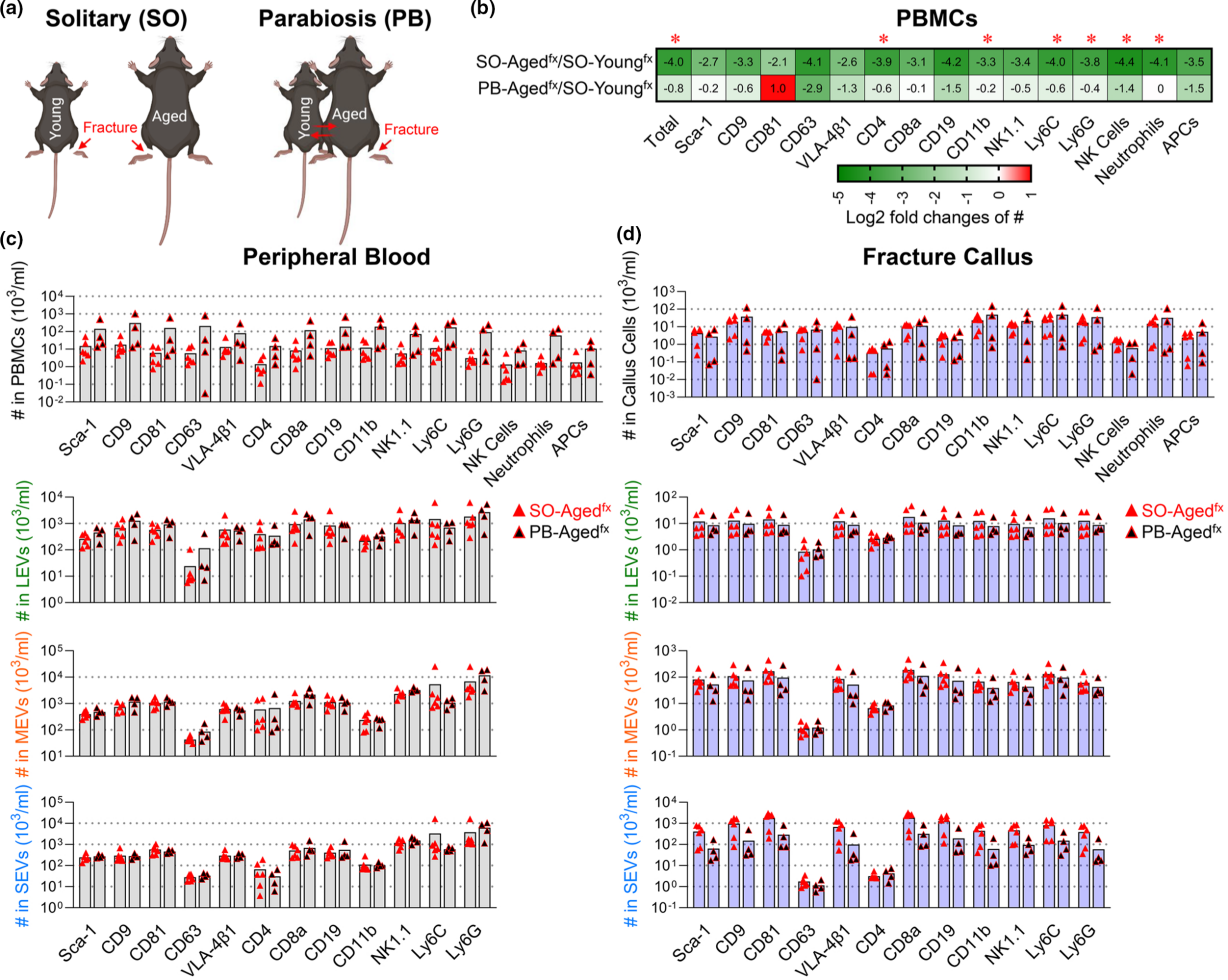
Heterochronic parabiosis (PB) increased the number of neutrophils and their associated plasma EVs in aged^fx^ mice. (a) PBMCs and plasma EVs were separated from solitary (SO) young^fx^ (4 months old) and aged^fx^ (24 months old) mice, as well as heterochronic (young^intact^–aged^fx^, 4 and 24 months) anastomosed PB mice (*n* = 4 pairs) on D7 post‐fracture, and profiled with the indicated surface markers using high‐resolution multicolor flow cytometry. (b) The heat maps represent a summary of the relative number of PBMCs, LEVs, MEVs, or SEVs for each surface marker depicted as the log2 fold change of two groups: SO‐aged^fx^/SO‐young^fx^ mice or PB‐aged^fx^/SO‐young^fx^ mice. A 0 reflects an equal number in the two groups; a positive log2 fold change reflects a higher number in the numerator group relative to the denominator group; and a negative log2 fold change reflects a lower number in the numerator group relative to the denominator group. The log2 fold changes were indicated as numbers in each cell of the heat maps. Comparisons between the two groups by an unpaired t test were performed with results indicated as *p < 0.05. (c, d) The graphs present a summary of the number of the different types of hematopoietic cells, and their associated LEVs, MEVs, and SEVs carrying each surface marker in peripheral blood (c) and fracture calluses (d) of SO‐aged^fx^ and PB‐aged^fx^ mice. Comparisons between SO‐aged^fx^ and PB‐aged^fx^ mice were performed using an unpaired t test; the differences did not reach statistical significance (*n* = 6 mice in each group)

We have previously demonstrated that heterochronic parabiosis rejuvenates aged bone fracture healing (Baht et al., 2015; Vi et al., 2018; Xiong et al., 2018). To investigate rejuvenation‐associated hematopoietic parent cell and EV profiles, we utilized our heterochronic parabiosis (PB) model (Figure 6a). Heterochronic parabiosis led to an accumulation of multiple hematopoietic cells in aged^fx^ mice by 7 days post‐fracture (Figure S4). Interestingly, the number of plasma EV subpopulations was similar in PB‐young^intact^ and PB‐aged^fx^ mice with generally small fold differences suggesting an active trafficking of plasma EVs between the animals via the shared blood supply (Figure S4).

The number of hematopoietic cells in peripheral blood of solitary (SO)‐aged^fx^ mice was transformed to a younger phenotype by heterochronic PB. Specifically, the number of all tested hematopoietic cells from peripheral blood of SO‐aged^fx^ mice was lower than SO‐young^fx^ mice (log2 fold changes ranged from −2.1 to −4.4) on D7 post‐facture (Figure 6b). By contrast, the corresponding log2 fold changes of PB‐aged^fx^/SO‐young^fx^ mice were all compensated or reversed (log2 fold changes ranged from −2.9 to +1; Figure 6b); this change was significant for total PBMCs, CD4^+^, CD11b^+^, Ly6C^+^, and Ly6G^+^ cells, neutrophils, and NK cells, indicating that provision of youthful blood components to aged fractured mice by heterochronic PB led to higher numbers of immune cells in PB‐aged^fx^ mice.

Compared with SO‐aged^fx^ mice, the peripheral blood of PB‐aged^fx^ mice (7 days post‐fracture) contained higher numbers of all tested hematopoietic cells (fold changes of PB‐aged^fx^/SO‐aged^fx^ mice ranged from 5.98 to 37.03); among these cells, neutrophils were the most enriched cell population (37.03 fold higher; Figure 6c). Consistent with cell‐level results, the number of neutrophil‐associated Ly6G^+^ EVs of all sizes was also higher in PB‐aged^fx^ than in SO‐aged^fx^ mice (Figure 6c). Our results indicate that the low circulating neutrophil number of aged mice, particularly with fracture, can be partially rejuvenated by youthful blood components supplied during heterochronic parabiosis to aged mice.

The number of all tested hematopoietic cells was higher in PB‐aged^fx^ calluses than SO‐aged^fx^ calluses 7 days post‐fracture (with the exception of Sca‐1^+^ and CD19^+^ cells); similar to peripheral blood, neutrophils were also the most enriched cell population in fracture calluses of PB‐aged^fx^ mice (Figure 6d). Interestingly, the numbers of all hematopoietic cell‐associated EVs were lower in the calluses of PB‐aged^fx^ relative to SO‐aged^fx^ mice, with the exception of CD4^+^ EVs, which were slightly higher (Figure 6d).

## 3 DISCUSSION

In this study, we observed an immunosenescence phenotype of aged mice, which was accentuated by the physical stressor of fracture injury and associated with impaired tissue regeneration. Decreased cell proliferation and/or increased cell death in aged animals was associated with decreased SEVs and increased LEVs in the circulation. This is especially pertinent as we and others have demonstrated a critical role for immune cells in bone fracture healing (Baht et al., 2018; Slade Shantz et al., 2014). In rodent models, an age‐associated dysregulation in inflammatory response to fracture injury is well documented (Baht et al., 2018) as we previously reviewed. Although aging has been associated with delays in healing in humans, there are little direct clinical data isolating the effects of aging on bone healing from the associated comorbidities that are frequently present in older adult populations (Meinberg et al., 2019). The increased risk of premature death after fracture in the geriatric population confounds the ability to compare rates of fracture healing in older and younger adults due to survivor bias. Nevertheless, age‐related changes affect many of the biologic processes involved in fracture healing in humans (Clark et al., 2017), and some studies demonstrate a decline in rate of fracture healing with age in humans (Dailey et al., 2018). Correction of this response by treatment with small molecules and/or cells leads to improved fracture healing in animals (Huang et al., 2020; Vi et al., 2018). Blood sharing with young animals by heterochronic PB increased the number of neutrophils in both peripheral blood and fracture calluses and their associated plasma EVs, in association with improved fracture healing in aged^fx^ mice.

Here, we identify that age greatly influenced the immunosenescence phenotype of parent cells and their associated EVs. Aged mice exhibited immunosenescence, as evidenced by significantly lower proportions of lymphoid lineages including CD11b^+^Ly6C^intermediate^Ly6G^high^ neutrophils, CD4^+^ T cells, CD11b^+^, NK1.1^+^ NK cells, and other CD63^+^ PBMCs (such as platelets and basophils). Taken together with higher percentages of HSCs in total PBMCs (identified based on their surface marker expression of Sca‐1, CD9, CD81, and VLA‐4β1), our findings are consistent with previous reports in aging that long‐term HSCs have increased self‐renewal and diminished lymphoid potential leading to a reduced capacity to differentiate into lymphoid lineages (Rossi et al., 2005).

Immunosenescence and inflammaging are associated with apoptosis of immune cells (Ginaldi et al., 2005). During apoptosis, parent cells disassemble into apoptotic bodies, a type of LEV (Budnik et al., 2016), which provides a plausible explanation for our observation that LEVs increased with age. Here, we also identified increased levels of pro‐inflammatory factors in LEVs associated with age, indicating their likely involvement in inflammaging (Fulop et al., 2017). We found significantly higher percentages of IFN‐γ^+^ and IL‐13^+^ PBMCs and a dramatically higher number of LEVs carrying all tested cytokines in aged mice than in young mice.

Different types of EVs have different effects. For instance, some EVs mediate cell‐to‐cell communication by transferring signaling molecules from their cells of origin to other cells. Some types of EVs are associated with adverse biological effects such as paracrine senescence (Borghesan et al., 2019; Takasugi, 2018), in part through carriage of senescence‐associated secretory protein cargo (Kadota et al., 2018). In contrast, EVs may also be associated with beneficial effects such as immune modulation or may serve as immunotherapeutic alternatives to stem cell therapies (Wiklander et al., 2019).

Here, we identified different types of EVs that could reflect an age‐dependent modulation of their parent cells or EV subtype and their cytokine content. The cytokine content was low in SEVs but high in LEVs in both young and aged mice; furthermore, the number of LEVs carrying cytokines was significantly higher in aged mice than in young mice. Thus, it is clear that EV subtype and age can impact EV cargo constituents and thereby the biological effects of EVs.

Fracture healing is one of the major challenges to physical resilience in older populations (Burge et al., 2007; Parker et al., 2020; Whitson et al., 2016). However, the predictors and mechanisms of age‐related changes in physical resilience remain undefined. Bone regeneration consists of four continuous and overlapping stages involving inflammation, development of a cartilaginous callus, development of a boney callus, and eventual remodeling of the healing tissue (Marsell & Einhorn, 2011; Vi et al., 2018). All of these stages involve communication between cells of the immune system and mesenchymal cells (Vi et al., 2018). Hematopoietic cells appear throughout these different phases of bone fracture healing to direct mesenchymal cell differentiation and activity in bone. In aged^fx^ mice, we observed dynamic changes in hematopoietic cell and EV profiles in the first week after fracture (most notably acute declines of multiple EV subtypes in contrast to acute increases in young mice) in association with impaired fracture healing (delayed bone deposition at tibial fracture sites with lower BV and BV/TV ratio within the fracture calluses). As a stressor, fracture injury accentuated the immunosenescence phenotype of aged but not young mice; compared with unfractured young mice, fracture injury of young mice did not significantly affect the number of hematopoietic cells, but induced secretion of multiple immune cell‐associated MEVs and SEVs.

Previous studies reported that MEVs and SEVs are fundamental elements in the inflammatory stage of fracture healing (Liu et al., 2019). SEVs from ASCs, MSCs, and bone marrow stem cells have been shown to have beneficial effects on fracture healing and bone regeneration (Li et al., 2018; Liu et al., 2019; Zhai et al., 2020; Zhang et al., 2016). However, more work is needed to understand the role of endogenous EVs during each stage of bone fracture healing. In addition, fracture injury triggered immune cells to produce other MEVs and some SEVs in young^fx^ mice, supporting the hypothesis that they contribute to fracture healing and bone regeneration. In contrast, in aged mice, the number of multiple immune cells, including neutrophils, was lower in fractured than in intact mice. Post‐fracture, especially during early stages of healing, the number of all major immune cell subtypes (with the exception of CD81) was significantly lower in aged^fx^ than in young^fx^ mice. In addition, the number of SEVs and MEVs carrying most of these surface markers was lower, while every LEV subtype was significantly higher in aged^fx^ mice. The decline of these stem cell‐ and immune cell‐associated plasma MEVs and SEVs after fracture in aged^fx^ mice may contribute to the defective fracture healing in aged subjects.

Soon after injury, neutrophils are recruited to the fracture site and secrete growth factors and cytokines leading to revascularization and recruitment of mesenchymal progenitor cells (Baht et al., 2018). Studies have reported neutrophils to be the most abundant immune cell population in the early fracture hematoma. Moreover, eliminating neutrophils by Ly‐6G‐Ab treatment impaired fracture healing in a mouse fracture model (Bastian et al., 2016; Kovtun et al., 2016). Beyond this apparent role within the fracture callus, neutrophils serve numerous functions in tissue regeneration. For instance, neutrophil‐derived microparticles contain functionally active endogenous anti‐inflammatory protein annexin 1 that results in an anti‐inflammatory response leading to a reduction in the recruitment of neutrophils (Dalli et al., 2008) and regulation of macrophage (Gasser & Schifferli, 2004) and dendritic cells (Eken et al., 2008). Efferocytosis of apoptotic neutrophils by macrophages can also modulate macrophages from a pro‐inflammatory to an anti‐inflammatory phenotype (Ferracini et al., 2013). We confirmed that neutrophils are highly enriched in fracture calluses compared with peripheral blood. We also observed that neutrophil numbers were lower in peripheral blood of aged^fx^ than in that of young^fx^ mice. Compared with SO‐aged^fx^ mice, the blood‐sharing of heterochronic PB resulted in a rescued immunosenescence phenotype in PB‐aged^fx^ mice characterized by high enrichment of neutrophils in both peripheral blood and fracture calluses and elevations of their associated Ly6G^+^ plasma EVs. It remains to be determined whether these particular EV subpopulations contribute directly to the improved bone fracture healing of parabiont aged^fx^ mice (Baht et al., 2015; Vi et al., 2018; Xiong et al., 2018).

Regenerative processes require efficient signal transduction among multiple cell types to orchestrate communication between cells of the immune system and the local tissue environment. EVs likely serve to transport these messaging molecules both systemically and locally (Liu et al., 2019). We previously identified a biomarker measure of physical resilience after hip fracture—the expected recovery differential—that captures the difference between actual recovery and predicted recovery in older adults. This biomarker explained 37% of the variance in recovery and included circulating metabolites, an inflammatory factor, and miRNA (Parker et al., 2020). In theory, each of these types of analytes can be carried by EVs but it remains to be determined whether any of these factors are EV‐borne and play a role in fracture healing.

While there are many methods for EV separation, there is still no “gold standard” separation method that applies to all downstream applications. We used polymer‐based precipitation to separate EVs from a small volume of plasma, and ultrafiltration to separate EVs from large volume cell‐free supernatants of digested calluses; both methods are among the most frequently used methods for EV separation with several advantages, including but not limited to the following: They are simple procedures with high yield suitable for large sample size studies, they preserve EV integrity, and they require only commonly used laboratory equipment. The limitation of both methods is the potential for coprecipitation of non‐EV contaminants (Carnino et al., 2019). In this study, although contaminants were limited, to decrease any influence of contaminants on our observations, we focused on EVs with the tested surface markers indicative of their cell origins. This study aimed to evaluate the responses of hematopoietic cells, especially immune cells, and their associated EVs to aging and the stress of tibial fracture injury. We observed high heterogeneity of EV subpopulations indicating the need for future investigations on the biogenesis, cargo sorting and packaging, and functions among the different EV categories. This study covered all major immune cell populations that are altered in immunosenescence, while future studies can be extended to subsets of immune cell populations, such as CD8^+^CD28^−^ T cells, and cellular senescence in aging and resilience.

Within our investigations here, we comprehensively evaluated the levels of parent hematopoietic cells, their cell‐associated plasma EVs in all sizes, and their alterations with the stressor of fracture. The age‐associated loss of immune cells and enrichment for cytokine‐carrying LEVs may contribute to the defective fracture healing in aged animals. While we do not provide direct evidence that specific hematopoietic cell subtypes, or gain or loss of their associated EVs mediate fracture healing phenotype in aged mice, the ability to reverse specific cell and cell‐associated EV alterations with heterochronic parabiosis in association with normalization of fracture healing points to mediation effects by these cell subtypes and cell‐associated EVs. More precise identification of the EV mediators of improved bone fracture healing could enable the delivery of therapeutic molecules to promote resilience to stressors and could serve as biomarkers of aging, resilience, and regeneration (Conlan et al., 2017; Liu et al., 2019). Future work is needed to identify whether neutrophil‐associated EVs, whose aging‐related declines were dramatically increased by heterochronic parabiosis, are sufficient to promote fracture healing in aged mice. Moreover, these results warrant future studies infusing EVs of young mice derived from particular hematopoietic cells or EV subpopulations to aged mice to assess their functional impact on fracture healing. EV‐related therapeutic strategies are clearly clinically relevant as there are currently 16 human clinical trials completed or in progress using EVs to treat various conditions including ulcers, diabetes, cancers, stroke, and kidney disease (Wiklander et al., 2019).

## 4 EXPERIMENTAL PROCEDURES

### 4.1 Mouse models

All protocols were approved by the Duke Institutional and Animal Care and Use Committee. C57BL/6J wild‐type mice (stock No. 000664) were purchased from Jackson Labs. Young (4 months old) and old (24 months old) mice were used for the studies.

### 4.2 Tibial fracture surgery

Fractures were performed as previously described (Huang et al., 2020). Briefly, mice were anesthetized and the surgical area proximal to the knee was shaved and disinfected. Following an incision, a hole was drilled into the tibial plateau and a 0.7‐mm stainless steel pin was placed into the medullary cavity and cut flush with the tibial plateau. A tibial fracture was induced mid‐shaft using blunt scissors, and the incision was closed using wound clips. For analgesia, 0.5 mg/kg buprenorphine‐SR (sustained release) was administered subcutaneously at the beginning of the procedure.

### 4.3 Analysis of fracture callus

Fracture calluses were harvested at the indicated time point post‐fracture and fixed in 10% Zn‐formalin at room temperature for 5 days. μCT analysis was conducted using a Scanco vivaCT 80 (Scanco Medical) at a scan resolution of 8 μm. Calluses were scanned 1 mm proximal and 1 mm distal from the fracture site and assessed for TV and BV in mm^3^, and ratio of BV‐to‐TV (BV/TV). Fixed fracture calluses were decalcified using 12% EDTA, pH 7.4, cleared of EDTA, and embedded in paraffin. Sections were cut at a thickness of 5 μm and stained using Safranin‐O/Fast Green to visualize bone and cartilage. A minimum of five sections were used to conduct computer‐assisted histomorphometry analysis, and results are presented as an amount relative to the total area of the fracture callus.

### 4.4 Parabiosis surgery

Anastomosis was achieved as previously described (Xiong et al., 2018). Briefly, 3‐ and 23‐month‐old female mice were surgically attached and allowed to heal for 4 weeks at which time the aged mice underwent tibial fracture surgery.

### 4.5 Cell separation

Blood was collected from intact mice and fractured mice on D3, D7, D14, D21, and D28 post‐fracture, and centrifuged at 3000 rpm (845 *g*) for 15 min at 4°C to separate plasma. Plasma samples were aliquoted and frozen at −80°C until future analysis. PBMCs were incubated with ACK lysis buffer (Quality Biological) to lyse red blood cells; PBMCs were then washed twice with PBS for flow cytometric profiling as we previously reported (Zhang et al., 2019).

Calluses were dissected as close to the fracture edge as possible and washed with PBS. Calluses were cut into 1‐mm^2^ pieces and digested with collagenase type I (0.2 mg/ml, Gibco) on a shaker at 37°C for 2 h (Huang et al., 2020). Digested calluses were passed through a cell strainer to remove cell debris and clumps, followed by centrifugation at 135 *g* for 5 min at 4°C to separate supernatants and cells. Cell‐free supernatants from digested calluses were aliquoted and frozen at −80°C until future analysis. Callus cells were incubated with Red Blood Cell Lysis Buffer (Thermo Fisher Scientific) at room temperature for 5 min, and then washed and stained with fluorescence‐conjugated antibodies for flow cytometry analysis.

### 4.6 EV isolation

Plasma and cell‐free supernatants from digested calluses were completely thawed, then centrifuged at 2000 *g* for 10 min at 4°C to remove remaining debris. To standardize the procedure, 20 μl plasma per sample was utilized for separating EVs for each staining tube. EVs in plasma were precipitated using ExoQuick (System Biosciences), a polymer‐based precipitation, and resuspended in double‐filtered (df)PBS for flow cytometric profiling as reported in our previous study (Zhang et al., 2020; Zhang, Huebner, et al., 2021). To isolate EVs from callus by ultrafiltration, cell‐free supernatants from digested calluses were twice passed through 5KD Sartorius Vivaspin™ 2 Centrifugal Concentrators. EVs with sizes over 5KD (about 2 nm) were collected for flow cytometric profiling. Separated EVs were profiled for size, bilayer structure, surface markers, cargo mitochondria, and cytokines as described in our previous studies (Zhang et al., 2020; Zhang, Huebner, et al., 2021). In addition, we also verified the isolated EVs by TEM imaging and checked for non‐EV contaminants by flow cytometry.

### 4.7 TEM

TEM of isolated EVs was performed at the Center for Electron Microscopy & Nanoscale Technology at Duke University Medical Center. Isolated EVs were diluted 1:100 in distilled water and immediately adsorbed onto Formvar‐ and carbon‐coated grids (Electron Microscopy Sciences). They were negatively stained with 2% aqueous uranyl acetate and examined in a JEM‐2100Plus Electron Microscope at 120 kV (JEOL USA). Micrographs were recorded on a 43 MP NanoSprint camera (AMT Imaging).

### 4.8 High‐resolution multicolor flow cytometry

As previously described (Zhang et al., 2019, 2020; Zhang, Huebner, et al., 2021), cells and EVs were profiled for 12 surface markers (Sca‐1, CD81, CD9, VLA‐4β1, CD63, CD8a, CD4, CD11b, NK1.1, Ly6C, Ly6G [BD Biosciences], and CD19 [Thermo Fisher Scientific]) and 8 cytokines (IL‐13, IL‐1β, and IL‐6 [Thermo Fisher Scientific], and IL‐4, IL‐10, IFN‐γ, IL‐17A, and TNF‐α [BD Biosciences]). The cytokines were only detectable in fixed and permeabilized EVs, confirming that they were carried by plasma EVs as internal cargo (data not shown). Unstained cells and EVs, antibodies without EVs, and single antibody‐stained cells, EVs, and UltraComp™ eBeads (Thermo Fisher Scientific) were used as staining controls to determine the fluorescence background. EVs were also profiled for ApoA1 (Novus Biologicals) to check for non‐EV contaminants. We performed serial dilutions of plasma (7.5‐, 15‐, 75‐, and 150‐fold to determine the optimal conditions for avoiding swarm detection. Each of these dilutions yielded similar staining patterns, but decreased percentages of positive populations as a result of increased percentages of non‐EV particles with PBS dilution (data not shown). For these reasons, we selected 7.5‐fold sample dilutions for analyses in this study. The percentages and number of the cells and EVs expressing each tested molecule were determined using a BD LSRFortessa X‐20 Flow Cytometer with the BD FACSDiVa software (BD Bioscience). Flow cytometry data analysis was performed using FCS Express 5 software (De Novo Software). For EV acquisition, high‐resolution multicolor LSRFortessa X‐20 Flow Cytometer was set up as reported in our previous study (Zhang et al., 2020; Zhang, Huebner, et al., 2021). Size reference beads with green fluorescence in mean sizes of 100, 500, 800, 1000, 3000, and 6000 nm (Thermo Fisher Scientific, Bangs Laboratories) were used for size estimation as previously described (Zhang et al., 2020).

### 4.9 Statistical analysis

GraphPad Prism 8.0 software (GraphPad) was used for statistical analysis. Statistical analyses assessing differences between young and aged mice were performed using an unpaired t test (Figures 1, 2, 3, 4, 5, and S1). Comparisons between intact mice and fractured mice at each time point were performed using one‐way ANOVA with Holm–Sidak's multiple comparisons test (Figures 4 and 5). Fold changes were calculated to compare the relative proportions of cell and EV (SEV/LEV) number in young, aged, and aged/young mice. A positive fold change reflects a higher number of the numerator group relative to the denominator group; a negative fold change reflects a lower number in the numerator group relative to the denominator group. A *p* < 0.05 was considered statistically significant.

## AUTHOR CONTRIBUTION

XZ, GB, and VBK designed the study. XZ, GB, RH, YC, KM, and SEM performed the experiments. XZ and GB analyzed the data. XZ, GB, and VBK drafted the manuscript. YC contributed to data interpretation. All authors revised and approved the final version of the manuscript for submission.

## CONFLICT OF INTEREST

None declared.

## Supporting information


Figure S1

Figure S2

Figure S3

Figure S4

## Data Availability

The datasets used and/or analyzed during the current study are available from the corresponding author on reasonable request.
