# Supplementary figures and images for: Rejuvenation of neutrophils and their extracellular vesicles is associated with enhanced aged fracture healing
Source: Aging Cell. 2022 Jun 3;21(7):e13651. doi: 10.1111/acel.13651 (PMC9282841; doi:10.1111/acel.13651)

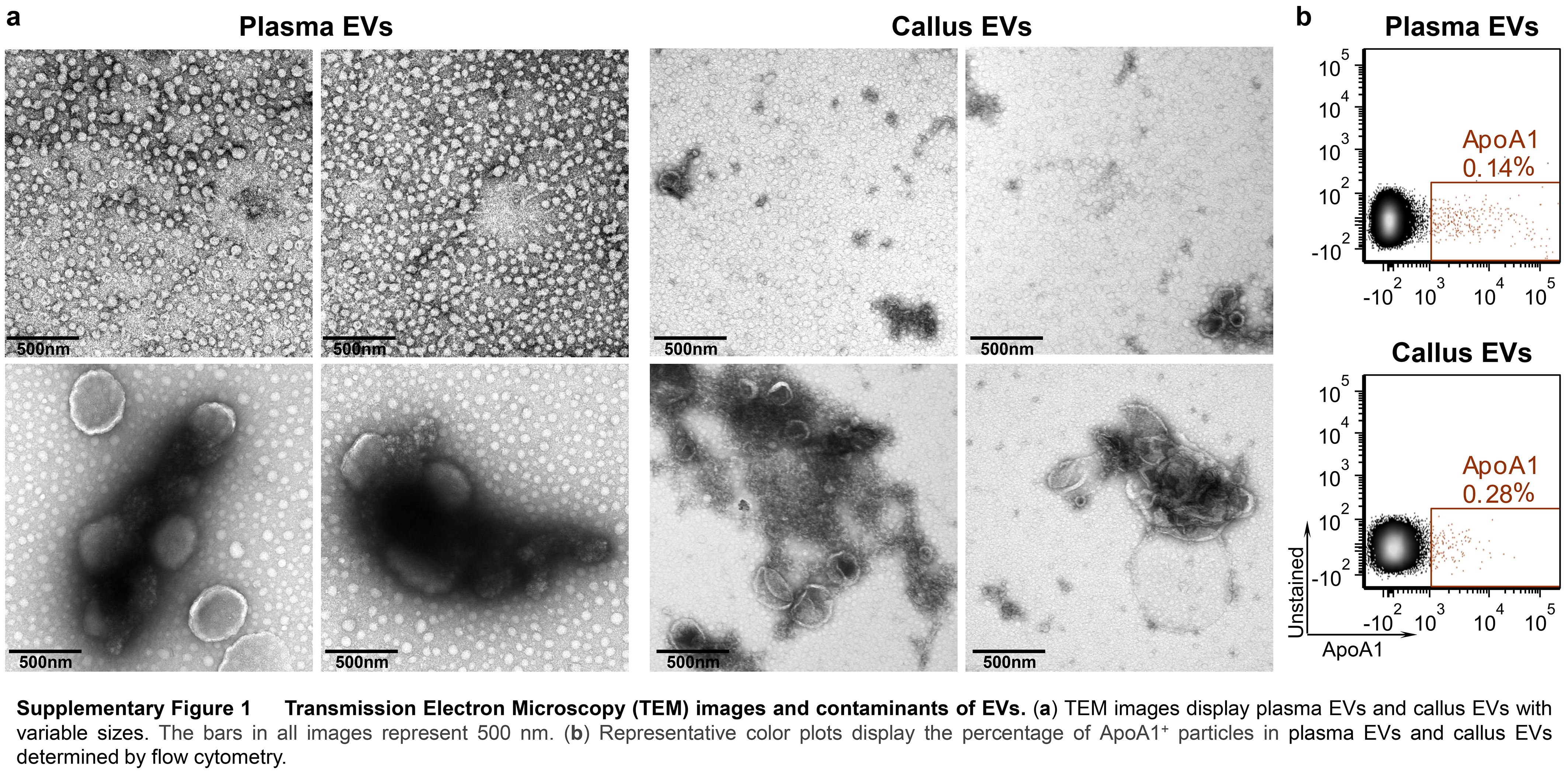

Supplement: Supplementary file 1 — Figure S1 [file ACEL-21-e13651-s001.tif]
