# Supplementary material for: Rejuvenation of neutrophils and their extracellular vesicles is associated with enhanced aged fracture healing
Source: Aging Cell. 2022 Jun 3;21(7):e13651. doi: 10.1111/acel.13651 (PMC9282841; doi:10.1111/acel.13651)
Supplement: Supplementary file 2 — Figure S2 [file ACEL-21-e13651-s003.pdf]

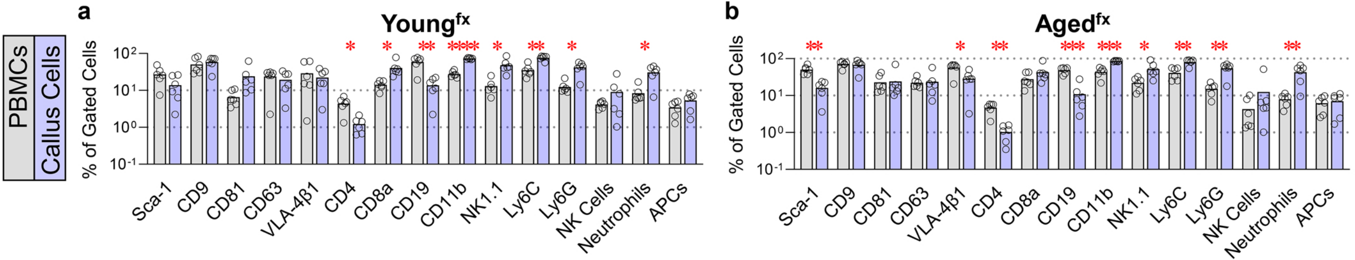

Supplementary Figure 2 Multiple immune cells were enriched in fracture calluses. PBMCs and fracture callus cells were harvested from young (age: 4 months) and aged (age: 24 months) mice (n=6 per group) on day 7 post fracture, and profiled with the indicated surface markers using high resolution multicolor flow cytometry. The graphs present a summary of the percentages of cell subsets expressing each surface marker in the matched PBMCs and callus cells of young-fractured (^{fx}) (**a**) and aged^{fx} (**b**) mice. Comparisons between the matched PBMCs and callus cells by paired t test for each marker were performed with results indicated as $*$ p <0.05, $**$p <0.01, $***$p <0.01, $****$p <0.0001.
