# Supplementary material for: Rejuvenation of neutrophils and their extracellular vesicles is associated with enhanced aged fracture healing
Source: Aging Cell. 2022 Jun 3;21(7):e13651. doi: 10.1111/acel.13651 (PMC9282841; doi:10.1111/acel.13651)
Supplement: Supplementary file 3 — Figure S3 [file ACEL-21-e13651-s004.pdf]

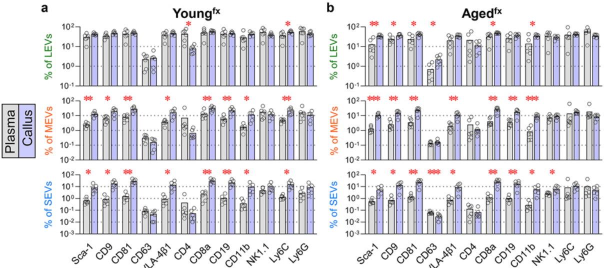

Supplementary Figure 3 Neutrophil-related EVs were enriched in fracture calluses. Plasma EVs and fracture callus EVs were harvested from young (age: 4 months) and aged (age: 24 months) mice (n=6 per group) on day 7 post fracture, and profiled with the indicated surface markers using high resolution multicolor flow cytometry. The graphs present a summary of the percentages of EV subpopulations carrying each surface marker in the matched plasma and calluses of young^{fx} (**a**) and aged^{fx} (**b**) mice. $CD11b^{+}Ly6C^{intermediate}Ly6G^{high}$ neutrophil-associated EVs include $CD11b^{+}$, $Ly6C^{+}$ and $Ly6G^{+}$ EVs. Comparisons between the matched plasma EVs and callus EVs by paired t test for each marker were performed with results indicated as * p <0.05, **p <0.01, ***p <0.01.
