# Supplementary material for: Rejuvenation of neutrophils and their extracellular vesicles is associated with enhanced aged fracture healing
Source: Aging Cell. 2022 Jun 3;21(7):e13651. doi: 10.1111/acel.13651 (PMC9282841; doi:10.1111/acel.13651)
Supplement: Supplementary file 4 — Figure S4 [file ACEL-21-e13651-s002.pdf]

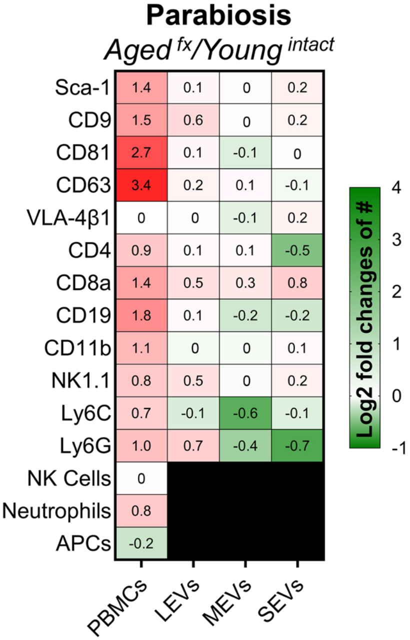

Supplementary Figure 4 A youthful blood supply led to an enrichment of multiple hematopoietic cells in the parabiosis (PB)-aged^{fx} mice. PBMCs and plasma EVs were separated from heterochronic (young^{intact}–aged^{fx}, 4 months and 24 months) anastomosed PB mice (n = 4 pairs) on D7 post fracture, and profiled with the indicated surface markers using high resolution multicolor flow cytometry. The heat maps represent a summary of the relative number of PBMCs, LEVs, MEVs or SEVs for each surface marker depicted as the log2 fold change of PB-aged^{fx} /PB-young^{intact} mice. A 0 reflects an equal number in the two groups; a positive log2 fold change reflects a higher number in PB-aged^{fx} mice relative to PB-young^{intact} mice; a negative log2 fold change reflects a lower number in in PB-aged^{fx} mice relative to PB-young^{intact} mice. The log2 fold changes were indicated as numbers in each cell of the heat maps.
